# Implementation of Liver Cancer Education Among Health Care Providers and Community Coalitions in the Cherokee Nation

**DOI:** 10.5888/pcd16.180671

**Published:** 2019-08-22

**Authors:** Behnoosh Momin, Jorge Mera, Whitney Essex, David Gahn, Margie Burkhart, Danielle Nielsen, Jennifer Mezzo, Alexander J. Millman

**Affiliations:** 1 Division of Cancer Prevention and Control, Centers for Disease Control and Prevention, Atlanta, Georgia; 2 Cherokee Nation Health Services, Tahlequah, Oklahoma; 3 Cherokee Nation Comprehensive Cancer Control Program, Tahlequah, Oklahoma; 4 ICF, Atlanta, Georgia; 5 Division of Viral Hepatitis, Centers for Disease Control and Prevention, Atlanta, Georgia

## Abstract

The Cherokee Nation Comprehensive Cancer Control Program collaborated with the Cherokee Nation Hepatitis C Virus (HCV) Elimination Program within Cherokee Nation’s Health Services to plan and implement activities to increase knowledge and awareness of liver cancer prevention among health care providers and the Cherokee Nation community. From August 2017 to April 2018, the 2 programs implemented liver cancer prevention interventions that focused on education of health care providers and community members. We used descriptive statistics to analyze data collected from a brief, retrospective pre–post survey for each intervention. We assessed overall awareness and knowledge of liver cancer and ability and intention to address it on a scale of 1 to 5. Project Extension for Community Healthcare Outcomes didactic sessions resulted in a 1.1-point improvement, provider education workshops resulted in a 1.4-point improvement, and presentations at community coalition meetings resulted in a 1.7-point improvement. Our study shows that HCV interventions can be used by public health and medical professionals interested in controlling HCV and related diseases such as liver cancer.

**Summary**

**What is already known on this topic?**

Chronic infections with hepatitis C virus (HCV) are risk factors for primary liver cancer. Lack of knowledge and awareness among health care providers, populations at high risk, and the public are barriers to HCV prevention and control.

**What is added by this report?**

The Cherokee Nation Comprehensive Cancer Control program and the Cherokee Nation Health Services HCV Elimination Program implemented and evaluated activities to increase knowledge and awareness. Overall, awareness, knowledge, ability, and intention increased among participants in the 3 interventions.

**What are the implications for public health practice?**

Provider and community education interventions can improve knowledge and awareness of liver cancer and the ability and intention to talk about it among health care providers and community coalitions.

## Introduction

Hepatocellular carcinoma (HCC), or primary liver cancer, is the second leading cause of cancer death worldwide among cancers that affect both men and women (1). In the United States, chronic infections with hepatitis B virus (HBV) or hepatitis C virus (HCV) are strong risk factors for HCC (2). Primary liver cancer incidence is increasing worldwide, including in the United States (3). Liver cancer is more common in men than in women, and among Asian/Pacific Islander, Hispanic, and American Indian/Alaska Native populations than in other racial and ethnic groups (4).

An Institute of Medicine report in 2010 described several barriers to HBV and HCV prevention and control efforts, including a lack of knowledge and awareness among health care providers, populations at high risk, and the public (5). The report included recommendations in 4 areas: improve viral hepatitis surveillance, improve provider and community education to increase knowledge and awareness of HBV and HCV, increase support for vaccine-based strategies to eliminate HBV transmission, and integrate and enhance viral hepatitis services, including risk factor screening and serologic testing.

The National Comprehensive Cancer Control Program (NCCCP), funded by the Centers for Disease Control and Prevention (CDC), provides funding and technical support for the development and implementation of cancer control programs to create cancer control plans in all 50 states, the District of Columbia, 8 tribes and tribal organizations, and 7 US territories (6). These plans guide the work of cancer control coalitions formed by each awardee. Coalitions include health department staff members (at the state, tribal, territory, US Pacific Island jurisdiction, and local levels) with expertise in cancer and their key partners, such as nonprofit organizations and community health centers. Awardee cancer control coalitions focus on current and emerging cancer issues in their target population and implement strategies in prevention, early detection, treatment, and survivorship by using policies, systems, and environmental changes to reduce the burden of cancer.

## Purpose and Objectives

In 2017, the Cherokee Nation Comprehensive Cancer Control (CNCCC) program initiated a partnership with the Cherokee Nation Health Services (CNHS) HCV Elimination Program. The goal of the HCV Elimination Program is to expand HCV testing and refer patients infected with HCV for treatment. The 2 groups collaborated to plan, implement, and evaluate activities to increase knowledge and awareness of HCC among health care providers and Cherokee Nation communities. Although lead staff members from the CNCCC program and HCV Elimination Program had collaborated on previous work, this partnership was their first official partnership. The objective of this study was to describe findings from the evaluation of 3 interventions implemented by the 2 programs. 

## Intervention Approach

The 2 programs implemented liver cancer prevention interventions from August 2017 through April 2018. Prevention strategies (Table 1) aligned with provider and community education recommendations in the 2010 Institute of Medicine report (5).

**Table 1:** Liver Cancer Education Interventions for Cherokee Nation Health Care Providers and Community Coalitions, 2017–2018

Intervention	Strategy	Description of Activity	No. of Completed Surveys/No. of Attendees	Participant Characteristics	Time in Practice
HCV Elimination Program	Conduct monthly Project ECHO^a^ didactic sessions	Conducted 15-minute didactic sessions during 8 Project ECHO clinics. Topics included incidence and prevalence of HCC, risk factors for and diagnosis of HCC, diagnosis of cirrhosis, and HCC surveillance.	72/83	All participants were health care professionals; 30.6% physicians; 20.8% nurses; 20.8% pharmacists; 8.3% psychologists; 6.9% nurse practitioners; 4.2% case managers; 8.3% described themselves as “other” health care professional	1 week to ≥40 years (median of 10 years in practice)
HCV Elimination Program	Conduct health care provider education workshops	Conducted 8 two-hour provider education workshops; one at a CNHS hospital, and 7 at CNHS outlying clinics. Topics included incidence and prevalence of HCC, risk factors for and diagnosis of HCC, diagnosis of cirrhosis, and HCC surveillance.	108/123	All participants were health care professionals: 35.2% nurses; 21.3% nurse practitioners; 17.6% physicians; 25.9% described themselves as “other” health care professional	0–50 years (median of 10 years in practice)
Cherokee Nation Comprehensive Cancer Control Program	Conduct presentations at community coalition meetings	Conducted 5 presentations at community coalition meetings, held in different venues in different geographic locations. Each presentation was approximately 30 minutes and was intended to reach the general community.	62/78	Participants represented 26 organizations	NA

Abbreviations: CNHS, Cherokee Nation Health Services; HCC, hepatocellular carcinoma; HCV, hepatitis C virus; NA, not applicable; Project ECHO, Project Extension for Community Healthcare Outcomes.

a Project ECHO is a collaborative model of education and care management that brings together health care providers to increase access to specialty treatment in rural and underserved areas (https://echo.unm.edu).

The HCV Elimination Program was responsible for conducting didactic sessions for CNHS health care providers on HCC epidemiology, diagnosis, and surveillance through the Project Extension for Community Healthcare Outcomes (ECHO) platform (The Echo Model). Launched in 2003 by a liver disease physician in Albuquerque, New Mexico, Project ECHO is a collaborative model of education and care management that brings together health care providers to increase access to specialty treatment in rural and underserved areas (7). The didactic sessions were delivered in a series of 15-minute presentations during regularly scheduled Project ECHO meetings that were hosted virtually using Microsoft Lync. The HCV Elimination Program was also responsible for conducting health care provider education workshops focused on liver cancer at 8 CNHS facilities. The same slide set used for Project ECHO didactic sessions was used for these workshops, but instead of delivering content in multiple sessions, the entire presentation was given in 1 workshop. The workshops were conducted in person at 7 of the 8 clinics and at the hospital in the Cherokee Nation in Oklahoma. Various health care providers, including nurses, case managers, physicians, nurse practitioners, and physician assistants, were invited to the workshops. 

The CNCCC program was responsible for making liver cancer prevention presentations to community coalition members at cancer coalition meetings. Content for each meeting was identical; however, content delivery was tailored to each audience. Presentations focused on causes, prevention, symptoms, and diagnosis of liver cancer. Participants represented 26 coalitions in the Cherokee Nation and included workers from the public school system, local recreation centers, farmers markets, and other community organizations. The CNCCC program presented liver cancer information at 5 coalition meetings.

## Evaluation Methods

For each of the 3 interventions, CNHS assessed changes among program participants in awareness, knowledge, ability, and intention by administering a brief, retrospective pre–post survey (8). Survey development was informed by materials developed by CNHS for provider and community presentations and outcomes of interest to CNHS. A paper-and-pencil survey was administered at the end of each session. For each intervention, we collected information on the number of participants and the number and types of medical professionals attending and organizations represented.

For didactic sessions and provider education workshops, *awareness* measured the provider’s awareness of statistics and the role of the liver and liver cancer; *knowledge* measured the provider’s knowledge of liver cancer risk factors, prevention, and signs and symptoms of the disease; *ability* measured the provider’s ability to identify patients who are at high risk for liver cancer, and *intention* measured the provider’s intent to speak with patients about HCC risk and recommend screening for patients at high risk. For the community coalition meetings, *awareness* measured the participant’s awareness of the function of the liver and liver cancer statistics; *knowledge* measured the participant’s knowledge of liver cancer risk factors, prevention, and signs and symptoms of the disease; *ability* measured the participant’s ability to speak with a health care provider about liver cancer risk and prevention, and *intention* measured the participant’s intent to speak with a health care provider about screening for HCV infection. Survey participants marked their responses on a Likert-type scale (from 1 to 5, with 5 indicating the best outcome).

Our analyses focused on assessing whether the interventions had any effect on participants’ awareness, knowledge, abilities, and intentions. Although pre-exposure and post-exposure data were reported in the same survey, surveys were completed anonymously; therefore, we were not able to match data for individual participants across didactic sessions. We combined items (awareness, knowledge, ability, and intention) to create a composite score for each intervention. We also developed an overall composite score, which used all survey questions pre-exposure and post-exposure to examine change overall. We calculated mean composite scores and standard deviations in Stata version 14 (StataCorp LLC). The range for the overall scores was calculated as an average of the ranges for all categories. The overall scores were calculated as an average of all of the scores that make up the score (awareness, knowledge, ability, intention). We used paired *t* tests to assess significance of overall change from pre-exposure to post-exposure (across awareness, knowledge, ability, intention) for each of the 3 interventions, but not for each variable of interest. We generated *P* values; however, not every individual attended all 8 didactic sessions. Therefore, differences between pre-exposure and post-exposure might not be large for the didactic sessions, which would cause the *P* values to be only slightly anticonservative. We excluded missing responses from analyses.

We collected contextual information about implementation challenges, facilitators, and lessons learned through ongoing communication between CNCCC and HCV Elimination Program staff members and NCCCP staff members. Lead CNCCC and HCV Elimination Program staff members took part in monthly technical assistance calls with NCCCP staff members throughout planning and implementation of the 3 interventions. At the close of the project, NCCCP staff members also met informally with lead staff members from each program to discuss final thoughts and experiences. 

## Results

Overall, awareness, knowledge, ability, and intention increased among participants in the 3 interventions. For overall awareness, knowledge, ability, and intention, Project ECHO didactic sessions resulted in a 1.1-point increase (2.9 pre-exposure vs 4.0 post-exposure, *t*_70_ = 3.02, *P* < .001), provider education workshops resulted in a 1.4-point increase (2.9 vs 4.3, *t*_101_ = 4.91, *P* < .001), and presentations at community coalition meetings resulted in a 1.7-point increase (2.5 vs 4.2, *t*_59_ = 4.3, *P* < .001). We found improvement in each variable of interest in each intervention (Table 2). The greatest improvement in knowledge and awareness (1.2-point increase for each) was in the Project ECHO didactic sessions. The provider education workshops had the greatest improvement in awareness (1.6-point increase), and the community coalition presentations had the greatest improvement in knowledge (2.1-point increase), closely followed by awareness (2.0-point increase).

**Table 2:** Pre-Exposure and Post-Exposure Composite Scores for Project ECHO^a^ Didactic Sessions, Health Care Provider Education Workshops, and Presentations at Community Coalition Meetings

Intervention/Variable of Interest	No. of Surveys Included in Analysis/No. of Completed Surveys	Composite Scores^b^	
		Pre-Exposure, Mean (SD) [Range]	Post-Exposure, Mean (SD) [Range]
**Project ECHO didactic sessions**			
Awareness of the role of the liver, liver cancer, and statistics	71/72	2.98 (1.02) [1–5]	4.14 (0.73) [1–5]
Knowledge of liver cancer risk factors, prevention, and signs and symptoms of the disease		2.72 (1.14) [1–5]	3.91 (0.80) [1–5]
Ability to identify at-risk patients		2.58 (1.05) [1–5]	3.70 (0.80) [1–5]
Intention to speak with patients about HCC risk and recommend screening for at-risk patients		3.05 (1.13) [1–5]	4.03 (0.88) [1–5]
Overall^c^		2.92 (1.03) [1–5]	4.03 (0.73) [1–5]
**Provider education workshops**			
Awareness of the role of the liver, liver cancer, and statistics	102/108	2.81 (0.81) [1–5]	4.39 (0.52) [1–5]
Knowledge of liver cancer risk factors, prevention, and signs and symptoms of the disease		2.96 (0.80) [1–5]	4.25 (0.65) [1–5]
Ability to identify at-risk patients		2.58 (1.05) [1–5]	3.70 (0.80) [1–5]
Intention to speak with patients about HCC risk and recommend screening for at-risk patients		3.05 (1.13) [1–5]	4.03 (0.88) [1–5]
Overall^c^		2.88 (0.81) [1–5]	4.25 (0.67) [1–5]
**Community coalition meetings**			
Awareness of the role of the liver and liver cancer statistics	60/62	2.23 (0.83) [1–4]	4.25 (0.54) [3–5]
Knowledge of liver cancer risk factors, prevention, and signs and symptoms of the disease		2.09 (0.98) [1–5]	4.20 (0.51) [3–5]
Ability to speak with a health care provider about liver cancer risk and prevention		2.23 (1.09) [1–5]	4.14 (0.66) [1–5]
Intention to speak with a health care provider about screening for hepatitis C virus infection		3.23 (0.99) [1–5]	4.23 (0.72) [1–5]
Overall^c^		2.49 (0.85) [1–5]	4.23 (0.59) [1–5]

Abbreviations: ECHO, Extension for Community Healthcare Outcomes; HCC, hepatocellular carcinoma; SD, standard deviation.

a Project ECHO is a collaborative model of education and care management that brings together health care providers to increase access to specialty treatment in rural and underserved areas (https://echo.unm.edu).

b Participants scored their awareness, knowledge, ability, and intention by using a Likert-type scale from 1–5, with 5 being the highest score for each variable measured.

c Paired *t* tests used to assess significance of overall change from pre-exposure to post-exposure (across awareness, knowledge, ability, intention) for each of the 3 interventions; *P* < .001 for each intervention overall.

Health care providers also improved their ability to identify patients at high risk for viral hepatitis and HCC and improved their intention to talk to patients about risk for the diseases. Among community coalition participants, we found an improvement in their ability and intention to talk to their health care provider about their risk for liver cancer and for getting tested for viral hepatitis.

## Implications for Public Health

Our study has implications for the cancer control community, including cancer control coalitions and health care providers, because our findings address awareness and education of health care providers, populations at risk, and the public about health risks associated with liver cancer. The study shows how provider and community education interventions can improve knowledge and awareness of liver cancer and the ability and intention to talk about it among health care providers and community coalitions. The improvements in each variable of interest and overall improvements were greater among participants in community coalition meetings than among participants in didactic sessions and provider education workshops. Although greater improvements might be attributed to initially lower levels of knowledge among community coalition participants compared with health care providers, improvements demonstrate the commitment of community coalition members in obtaining vital information needed to best address the needs of their target population.

Several factors facilitated implementation of our intervention, and awareness of these factors can help others in implementing similar interventions. Regularly scheduled meeting times (ie, through Project ECHO) with providers interested in liver cancer and who care for relevant patient populations increased participation rates and resulted in a captive audience and a robust discussion. A collaborative approach across programs facilitated access to essential evaluation resources and expertise. An established relationship with clinic directors (ie, infectious disease specialists through Project ECHO) helped in scheduling the workshops and ensuring provider participation. Involving CNHS public health educators in the coordination of coalition meetings ensured that liver cancer prevention presentations were included in meeting agendas and that space was secured for each meeting. Access to audiovisual equipment and printing resources and a meeting facilitator with a flexible schedule helped accommodate schedules of participants.

We found numerous challenges in planning and implementing these interventions. Developing the PowerPoint presentation for the Project ECHO didactic sessions and provider education workshops was challenging in scheduling, time consumption, and commitment. Finding convenient meeting times for the majority of health care providers was difficult. Also, competing responsibilities of CNHS public health educators made it difficult for them to dedicate a substantial amount of time to coordinating activities. For example, they traveled for 2 or 3 hours to reach community coalition meetings.

We learned several valuable lessons. The first is to start small. CNHS planned and implemented 3 unique liver cancer prevention interventions simultaneously. However, both NCCCP and HCV Elimination Program staff members found the workload demanding and in hindsight felt that focusing on just 1 or 2 activities at a time would have been better. Second is to be realistic about resources required, such as time and staffing. Planning and implementing the 3 interventions took a large amount of time. CNHS reported through interviews that it had to continuously shuffle priorities to conduct the provider education sessions and complete other time-intensive tasks. In addition, many administrative tasks (eg, monthly reporting requirements, scheduling workshops, completing data tracking sheets) were time consuming. The CNHS team provided an administrative staff member to complete these tasks. The third lesson is to identify and maximize resources. Both NCCCP and HCV Elimination Program staff members spent substantial time developing content for presentations. Greater collaboration among staff members in various programs and outreach to others who might have already developed high-quality resources that required only minor adjustments for the population of interest could have increased the efficiency of staff members and decreased the time required to implement the interventions.

Liver cancer rates are increasing in the United States and public health programs, including the NCCCP, should continue to build and diversify their work in addressing viral hepatitis for liver cancer prevention. The CNHS cancer control and HCV elimination programs have demonstrated a successful partnership model to address liver cancer that can be adapted by other programs. Increasing viral hepatitis and liver cancer prevention interventions among all public health programs is an important step in lowering liver cancer rates in the United States.

## References

[1] Ferlay J , Soerjomataram I , Dikshit R , Eser S , Mathers C , Rebelo M , Cancer incidence and mortality worldwide: sources, methods and major patterns in GLOBOCAN 2012. Int J Cancer 2015;136(5):E359–86. 10.1002/ijc.29210 25220842

[2] Di Bisceglie AM , Lyra AC , Schwartz M , Reddy RK , Martin P , Gores G , Hepatitis C-related hepatocellular carcinoma in the United States: influence of ethnic status. Am J Gastroenterol 2003;98(9):2060–3. 10.1111/j.1572-0241.2003.t01-1-07641.x 14499788

[3] Ryerson AB , Eheman CR , Altekruse SF , Ward JW , Jemal A , Sherman RL , Annual report to the nation on the status of cancer, 1975–2012, featuring the increasing incidence of liver cancer. Cancer 2016;122(9):1312–37. 10.1002/cncr.29936 26959385 PMC4840031

[4] US Cancer Statistics Working Group. United States cancer statistics: 1999–2015 incidence and mortality web-based report. Atlanta (GA): US Department of Health and Human Services, Centers for Disease Control and Prevention, and National Cancer Institute; 2018. http://www.cdc.gov/uscs. Accessed October 17, 2018.

[5] Committee on the Prevention and Control of Viral Hepatitis Infection, Institute of Medicine. Hepatitis and liver cancer: a national strategy for prevention and control of hepatitis B and C*.* Colvin HM, Mitchell AE, eds. Washington (DC): National Academies Press; 2010. 25032367

[6] Centers for Disease Control and Prevention. National comprehensive cancer control program. https://www.cdc.gov/cancer/ncccp/index.htm. Accessed October 30, 2018.

[7] Project ECHO, The University of New Mexico. A revolution in medical education and care delivery. https://echo.unm.edu. Accessed September 4, 2018.

[8] Pratt CC , McGuigan WM , Katzev AR . Measuring program outcomes: using retrospective pretest methodology. Am J Eval 2000;21(3):341–9. 10.1177/109821400002100305

